# Timely initiation of complementary feeding and associated factors among children aged 6 to 12 months in Northern Ethiopia: an institution-based cross-sectional study

**DOI:** 10.1186/1471-2458-13-1050

**Published:** 2013-11-06

**Authors:** Ashenafi Shumey, Meaza Demissie, Yemane Berhane

**Affiliations:** 1 Public Health Department, College of Health Sciences, Mekelle University, Mekelle, Ethiopia; 2 Addis Continental Institute of Public Health, Addis Ababa, Ethiopia

**Keywords:** Timely initiation, Complementary feeding, Ethiopia

## Abstract

**Background:**

Exclusive breastfeeding (EBF) for the first six months of life is critical for the wellbeing of the child. In the mean while, timely initiation and starting nutritionally-adequate, safe, age-appropriate complementary feeding at six months is recommended for the better health and development of infants. According to the Ethiopian Demographic and Health Survey 2011, timely initiation of complementary feeding in Ethiopia at the 6th month was only 51%. The purpose of this study is to determine the magnitude of timely initiation of complementary feeding and associated factors in Mekelle town, Northern Ethiopia.

**Methods:**

An institutional based cross-sectional study design was conducted among 422 mothers of infants aged from six months to one year selected from six public health facilities. Sample size proportional to the patient flow rate of each institution was allocated and systematic random sampling method was used to get the study participant. An exit interview using structured questionnaire was conducted about their experience on complementary feeding and related experience. The questionnaire was pretested among 21 mothers. Data were entered with EPI info version 3.5.1 and cleaning and analysis was done by using SPSS version 16. Frequencies distribution, binary and multiple logistic regressions were done. OR and 95% confidence interval was computed.

**Result:**

The prevalence of timely initiation of complementary feeding at sixth month was 62.8% (265/422, 95% C.I: 58.1, 67.31%). Educational level, occupation of mother, parity, having ANC follow up, and birth preparedness were found to be independent predictor of timely initiation of complementary feeding.

**Conclusions:**

Almost two-third of mothers initiated complementary feeding at six month of child’ age as recommended. This was relatively higher prevalence than most developing countries. However, significant proportion of mothers still did not initiate complementary feeding timely. Mothers who are illiterate and completed only primary school need more attention. All mothers must be encouraged to make antenatal care follow up.

## Background

Complementary feeding is giving infants other foods or fluids in addition to the breast milk at six months age of child [1]. Adequate nutrition during infancy and early childhood is critical to the development of children’s full human potential. Exclusive breastfeeding is critical and adequate for the first six months of child. However; after six months breast milk alone is no longer sufficient to meet late infant’s nutritional requirements and, therefore, timely starting nutritionally-adequate, safe, age-appropriate complementary feeding at sixth month of age is recommended for better health and development of infants [2-8]. According to the Ethiopian Demographic and Health Survey 2011, timely initiation of complementary feeding in Ethiopia at the 6th month was only 51% [9] which was 10% in Nairobi, 50% in Taiwan and 55.1% in West Bengal India [10-12].

The estimated infant mortality for Ethiopia have been 77 per 1000 live births for a long time, however, the current demographic and health survey reported a reduction in infant mortality which is 59 deaths per 1,000 live births [9]. It is also documented that poor infant feeding practice which is poor breastfeeding and poor complementary feeding contribute to 24% of infant deaths [13].

Poor complementary feeding practices means that many children continue to be vulnerable to irreversible outcomes of stunting, poor cognitive development, and significantly increased risk of infectious diseases leading to gastroenteritis, diarrhea and acute respiratory infection [7,14-16].

Exclusive breastfeeding for six months followed by timely initiation of appropriate complementary feeding with continued breastfeeding for the first year of life could avert 13 percent (1.3 million) of the more than 10 million deaths among children under 5 every year [17]. Regardless of the policy and strategies adopted by Ethiopian Ministry of Health, the change seen in the practice of the timely initiation of complementary feeding from 2005 to 2010 was insignificant.

The aim of this study was therefore: i) to determine the prevalence of the timely initiation of complementary feeding among 6 to 12 months age children in Northern Ethiopia; ii) to identify factors associated with the timely initiation of complementary feeding among 6 to 12 months age children in Northern Ethiopia.

## Methods

### Study area and period

This study was conducted in public health facilities found in Mekelle town, Northern Ethiopia. The town is the capital city of Tigray region and is located 783 Kilometers north of Addis Ababa, the capital of Ethiopia. Total population of Mekelle, as to 2007 census, was 177,090. Of the 9 public health facilities found in Mekelle zone, six public health facilities are found in the town in which this study was conducted. This study includes two hospitals, one regional and the other referral hospital, and 4 health centers which all give service for all the Mekelle town community members without restriction. The excluded facilities are, not providing service specifically for the town residents but to Mekelle zone, they were either newly built or found in areas recently included to Mekelle. The study was conducted from January to June 2011.

#### Study design, population and sampling

An institution based cross sectional was conducted. Mothers of infants aged from 6 months to one year in Mekelle town were the study population. The sample size for the study was calculated using single population proportion formula using the assumption that the proportion of mothers who initiate complementary feeding timely to their child to be 50%, 95% CI, 5% marginal error, and 10% none response rate, a total of 422 mothers were required for the study.

The sampling unit was taken from each, six, public health institutions based on their predetermined patient flow rate. To allocate the study subjects, first the average numbers of clients who visit the under five outpatient department daily was estimated by referring client registration book/record for two weeks prior to data collection. Proportional allocation was made based on the possible number of patients that would be expected during the study period and systematic random sampling was employed to identify study participants. Sampling interval was determined by dividing the expected patient with in the study period by allocated sample size to each facility.

As to the simple observation made to assess the difference in characteristics of clients of the health centers and hospitals, utilization of the services was according to the physical proximity of facilities. In fact referral cases from different institutions come to hospitals which are not included in this study. Based on this fact, all institutions were considered as homogeneous.

### Data collection

Data were collected by client exit interview from all mothers of infants aged between 6 months to one year attended under five clinics in all governmental health institution found in Mekelle town using structured questionnaire. Structured questionnaire which was originally prepared in English then translated to local language Tigrigna. The questionnaire was developed and refined on the basis of contents of peer review journals. Six female extension nursing students were recruited to collect data during working hours of the health institutions for the whole data collection period.

Data quality control; Structure questionnaire was translated to local language and then back to English by two people for consistency. Pretest was made in 21 (5%) mothers, in the study area, which was not included in the study to assess the content and approach of the questionnaire. One supervisor was recruited and training was given to data collectors and supervisor for two days on the objective, relevance of the study, confidentiality of information, respondent’s right, about pre-test, informed consent and techniques of interview; after the investigators discuss deeply.

### Data analysis, presentation and interpretation

Data were entered using EPI info version 3.5.1 statistical software and cleaning and analysis was made using SPSS version 16. Cleaning was made using frequencies. Univariate was done to describe dependent and independent variables; percentages, frequency distributions and measures of central tendency and measures of dispersion were used for describing data. Then binary logistic regression was made to see the crude significant relation of each variable with dependant variables. Finally, independent variables found significant were entered to multivariate logistic regressions to control the effect of confounding. Stepwise backward LR was used for multiple logistic regressions. Odds ratio with 95% confidence interval to ascertain association between independent and dependent variable was used.

#### Ethical considerations

Ethical clearance was obtained from institutional review board of Addis Continental and Mekelle University. The offices of Tigray Region Health Bureau and Mekelle zonal health Department, the selected Health institutions were communicated with formal letters from the Mekelle University.

All the study participants was reassured that they would be anonymous. Names or any personal identifiers were not recorded. Respondents were clearly told about the study and the variety of information needed from them. They were given the chance to ask anything about the study and made free to refuse or stop the interview at any moment they want if that was their choice. After interview the importance of timely initiation of complimentary feeding was discussed with the respondents who had not had knowledge on complementary issues.

## Results

### Socio-demographic characteristics of the mothers

A total of 422 women having infant aged six months to one year were included in the study. There was 100% response rate. The overall mean age was 26.34 ± 5.48 (SD) with the range of 29 (16, 45). Majority of the respondents, 315 (74.6%) were able to read and write. More than half of the mothers, 54.3%, were unemployed. Three hundred twenty eight (77%) were either married or were living with their partner. The vast majority of the respondent mothers, 363 (86%), were orthodox Christians and more than 95%of the mothers were Tigrean in ethnicity (from the same region). Husbands of 83% (308/371) were employed or working for private sector; only 3(0.8%) were unemployed. The median family size was 4. Among all respondents, 286 (67.8%) had television and 310 (73.5%) mothers had radio. Approximately 54% of the respondents (229/422) had both television and radio (Table 1).

**Table 1:** Socio-demographic and medical history of mothers, who had infant aged from six months to one year, attended public facilities in Mekelle town, from December 2010 – January 2011

**Variables**	**Category**	**Frequency (n)**	**Percentage**
Age of the mother	15–19	29	6.9
	20–24	141	33.4
	25–29	133	31.5
	30+	119	29.2
Educational status of women	Illiterate	112	26.5
	1–4	25	5.9
	5–8	93	22.0
	9–10	104	24.7
	11+	88	20.9
Husband education (n = 324)	Illiterate	98	23.2
	1–4	15	3.6
	5–8	73	17.3
	9–10	86	20.4
	11+	150	35.5
Number of pregnancy	1–2	275	65.2
	3–5	113	26.8
	6+	34	8.1
Months of pregnancy during Initiation of ANC (n = 368)	1–3	113	30.7
	4+	255	69.3
Frequency of ANC (n = 368)	1–4	254	69.0
	5+	114	31.0
Place of birth	Home	87	20.6
	Public facility	313	74.2
	Health post	4	0.9
	Private clinic	18	4.3
Individual who assisted the delivery	TBA/TTBA	20	4.7
	Mother	59	14.0
	Relatives	19	4.5
	HEWs	7	1.7
	Health professional	317	75.1

### Medical history of the mothers

Three hundred sixty eight (87%) mothers had antenatal care follow up at least once during the last pregnancy. About 70% (294/422) of mothers had at least 4 visits as recommended. Near to 80% of all mothers had institutional delivery and 87 (20.6%) mothers reported home delivery. About 70% (294/422) of mothers had received postnatal care (Table 1).

### Breast feeding pattern

Almost all of respondent mothers, 418 (99.1%), had initiated breast feeding and this decreased to 410 (97.2%) at the six month and majority, 96.4%, were still breast feeding at the time of study. Only 15 (3.6%) mothers had stopped breastfeeding before the child’s sixth months of age.

Very few, only 4, mothers did not initiate breast-feeding at all; the main reasons were no breast milk (2), breast disease (1) and having HIV/AIDS (1). The supplemented foods were cow’s milk and formula diet.

### Complementary feeding

Of all mothers who had breastfeeding experience, 391 (93.5%, n = 418) had started complementary feeding at the time of the interview with a range of 2 weeks to 11 months. Of all 19.7% (83/422) of mothers initiated complementary feeding before the age of six months. And about 17.54% (74/422) mothers had initiated complementary feeding beyond six months. However, the prevalence of timely initiated complementary feeding at the age of six months was 62.8% (265/422, 95% C.I: 58.1, 67.31%). The majority of the respondents 310 (73.5%) knew that a child was supposed to be exclusively breastfed for only six months, however, 48 (11.4%) could not be sure about the right duration of exclusive breastfeeding and the other 64 (15.1%) had a wrong idea about the right time of exclusive breast feeding.

The complementary food given by most of mothers was cereal based fluids (46.5%, 181/389) followed by cow’s milk (20.1%, 78/389), formula milk (11.3%, 44/389), family diet (8.7%, 34/389) and the rest (13.4%, 52/389) used the combination of two or more the above foods. Approximately 25% of mothers had used bottle feeding either consistently or sometimes.

#### Factors found associated with timely initiation of complementary feeding at sixth month

After applying bivariate and multiple logistic regressions, five variables were found to be significantly associated with timely initiation of complementary feeding. These were education of mothers (greater than grade nine (AOR = 2.361)), mothers who were housewife or unemployed (AOR = 2.347)), mothers had at least one child (AOR = 2.344), and those mothers who made ANC follow up (AOR = 2.845) and birth preparedness (AOR=2.816) were more likely to initiate complementary feeding timely at sixth month (Table 2).

**Table 2:** Factors associated with timely initiation of complementary feeding among mothers who had infant aged from six months to one year, attended public facilities in Mekelle town, from December 2010 – January 2011

**Variable**	**Category**	**Complementary feeding timely**		**COR**^**a **^**(95% CI)**	**AOR**^**b **^**(95% CI)**
		**Yes**	**No**		
Age of mothers	<=19	13	16		
	20–29	172	102	2.075 (0.959, 4.490)	0.972 (0.374, 2.524)
	>30	80	39	2.525 (1.105, 5.766)	1.764 (0.578, 5.377)
Educational level of mother	Illiterate	54	58		
	1–8	68	50	1.461 (0.868, 2.458)	0.933 (0.478, 1.818)
	9–12+	143	49	3.135 (1.915, 5.130)	2.361 (1.144, 4.869)
Occupation of mother	Employed	111	82		
	Jobless(housewives)	154	75	1.517 (1.020, 2.256)	2.347 (1.404, 3.921)
Marital status	Not in union	40	11		
	Married/in union	254	117	7.894 (3.911, 15.934)	1.57 (0.547, 4.505)
Educational status of husband	Illiterate	32	66		
	1–8	56	32	3.609 (1.970, 6.614)	1.901 (0.941, 3.839)
	9–12+	177	59	6.187 (3.698, 10.354)	2.991 (1.501, 5.957)
Television ownership	No	60	76		
	Yes	205	81	3.206 (2.096, 4.904)	0.969 (0.512, 1.834)
Radio ownership	No	54	58		
	Yes	211	99	2.289 (1.471, 3.558)	1.444 (0.854, 2.442)
Number of live birth	1	100	78		
	>1	165	79	1.629 (1.093, 2.429)	2.344 (1.438, 3.822)
ANC follow up	No	13	41		
	Yes	252	116	6.851 (3.536, 13.276)	2.845 (1.240, 6.528)
Postnatal care	Yes	67	61		
	No	198	96	1.878 (1.229, 2.869)	0.860 (0.492, 1.505)
Planned child	No	55	57		
	Yes	210	100	2.176 (1.401, 3.381)	1.052 (0.589, 1.885)
Place of delivery	Out of health facility	40	51		
	Health facility	225	106	2.706 (1.685, 4.348)	0.720 (0.270, 1.920)
Assistant of the delivery	Non professional	48	57		
	Professional	217	100	2.577 (1.641, 4.046)	1.253 (0.690, 2.275)
Birth preparedness	No	22	57		
	Yes	243	100	6.296 (3.653, 10.850)	2.816 (1.459, 5.437)
Knows optimal BF	No	175	130		
	yes	90	27	2.476 (1.523, 4.027)	1.426 (0.806, 2.522)

^a^COR Crude Odds Ratio.

^b^AOR Adjusted Odds Ratio.

## Discussion

The result of our study revealed that timely initiation of complementary feeding is 62% with the median age of six months. House wife mothers, mothers and husbands education, ANC attendance, parity and birth preparedness were the factors found to increase initiation of complementary feeding on time.

The 62.8% prevalence we detected was higher than the national prevalence (51%) [8]. It is also much higher than the study from Nairobi, (less than 10%), Taiwan (50%) and West Bengal, India (55.1%) [10-12]. Our finding, although it is better than the one reported at national level and few other countries, it was still low as the remaining 38% of mothers did not start complementary feeding at the right time. This relatively higher prevalence of timely initiation of complementary feeding at six month can be explained by the expansion of the health extension workers in Ethiopia. The health extension workers among their other health activities are involved in continuous health education to the community which has contributed to high coverage of ANC attendance and health institutional delivery and also initiation of complementary feeding on time in Mekelle town. The median age of starting complementary feeding was six month, where as in a country like Ghana they started earlier at 5.4 months of the child age [18].

Not few mothers (8.7%) used family diet as a complementary feeding while solid foods are not recommended at this age of infancy. Furthermore, majority of mothers used fluids as a complementary food that might dilute the nutritional contents and affect growth and development of children. Approximately 18% of children started complementary feeding late at seventh month or beyond, which would lead to macro and micro nutrient deficiency and serious nutritional problem, slows growth and predisposes to infectious disease that further exacerbates the nutritional status [7].

Mothers who had at least one child were 2.344 times higher in initiating complementary feeding at six months than mothers who had only one child, which is congruent with findings in other places [19]. Studies revealed that mothers who had at least one child have high levels of breastfeeding related knowledge, including appropriate time of initiating complementary feeding [20]. This may also be related to repeated exposure of health education during the previous pregnancies and the experience they gained through time, therefore repeated exposure to health education will have an impact in subsequent pregnancies.

A higher maternal educational level, high school and above (AOD = 2.361), was noted to increase timely initiation of complementary feeding; similar findings were observed by other studies in Nairobi, Kenya [15], Hong Kong [19], Belgium [21], and Bavaria, Germany [22]. This can be explained that improved maternal education enhances mothers’ understanding and appreciation of the demands and benefits of introducing complementary feeding timely, and empowers them to resist external interferences and pressures.

A higher husband educational level, high school and above (AOD = 2.991), was also noted to favor timely initiation of complementary feeding as education is believed to enable the husbands to understand their wives and provide help and approval to what mothers would like to do to keep the child healthy.

Antenatal care attendance seems to have an impact, AOR = 2.845, on exclusive breastfeeding for the first six months and initiating complementary feeding at the six months. Therefore, ANC is very important to also deliver other maternal health related messages to women.

House-wife mothers were more likely to initiation of complementary feeding timely which was in line with finding in other places [22]. This can be because the housewives usually stay at home and would not be obliged like mothers who are working to wean early to go to work.

Mothers who made birth preparedness were 2.816 times more likely to initiate complementary feeding at six month than those who do not. This might enable mothers to arrange conditions which could help them initiate complementary feeding timely.

Though, some other findings revealed that being older age is favorable to initiate complementary feeding timely [19,21,22], in this study age had no association with timely initiation of complementary feeding, similar to the finding from Nigeria [23].

## Conclusion

Almost two-third of mothers initiated complementary feeding at sixth month of child age that is relatively higher prevalence than other countries. In order to reduce the high infant mortality this level has to be increased since 38% of the mothers are still not starting complementary feeding at six months. Education status of the mother and the husband, occupation of the mother, number of children, antenatal care follow up and birth preparedness are the predictors of timely initiation of complementary feeding.

We recommend,

• The high ANC attendance in this area is also accompanied with high institutional delivery and timely imitation of the complementary feeding unlike other places areas within the country. Therefore, it is important to further analyze what contributed to this achievement in this area so that it can be tried in other parts of the country.

• The mass media could result in positive changes if child feeding messages were transmitted; as all are urban women, who has at least one means of source of information (Radio or/and TV) and are more likely to listen to and be influenced by it.

• Mothers who are illiterate and completed only primary school need more attention.

• All mothers must be encouraged to make antenatal care follow up and birth preparedness.

## Competing interests

The authors declare that they have no competing interests.

## Authors’ contributions

AS: Initiated the research, wrote the research proposal, conducted the research, did data entry and analysis and wrote the manuscript. MD: Involved in the write up of the proposal, write up of the manuscript. YB: Involved in the write up of the proposal, write up of the manuscript. All authors read and approved the final manuscript.

## Authors’ information

Ashenafi Shumye: BSc, MPH.

Meaza Demissie: MD, MPH, PhD, Assoc. Prof. Public Health, at Addis Continental institute of public Health.

Yemane Berhane: MD, PhD, Prof. of Epidemiology and Public Health, at Addis Continental institute of public Health.

## Pre-publication history

The pre-publication history for this paper can be accessed here:

http://www.biomedcentral.com/1471-2458/13/1050/prepub
